# CRISPR/Cas9‐mediated mutations of *FANTASTIC FOUR* gene family for creating early flowering mutants in tomato

**DOI:** 10.1111/pbi.14223

**Published:** 2023-11-09

**Authors:** Lele Shang, Jinbao Tao, Jianwen Song, Yaru Wang, Xingyu Zhang, Pingfei Ge, Fangman Li, Haiqiang Dong, Wenxian Gai, Donald Grierson, Zhibiao Ye, Yuyang Zhang

**Affiliations:** ^1^ National Key Laboratory for Germplasm Innovation and Utilization of Horticultural Crops Huazhong Agricultural University Wuhan 430070 China; ^2^ Hubei Hongshan Laboratory Wuhan 430070 China; ^3^ Plant Sciences Division, School of Biosciences University of Nottingham Loughborough LE12 5RD UK; ^4^ Shenzhen Institute of Nutrition and Health Huazhong Agricultural University Wuhan 430070 China; ^5^ Shenzhen Branch, Guangdong Laboratory for Lingnan Modern Agriculture, Genome Analysis Laboratory of the Ministry of Agriculture, Agricultural Genomics Institute at Shenzhen Chinese Academy of Agricultural Sciences Shenzhen 518000 China

**Keywords:** CRISPR/Cas9, early flowering, *FANTASTIC FOUR*, germplasms, tomato

## Abstract

Flowering time is of great agricultural importance and the timing and extent of flowering usually determines yield and availability of flowers, fruits and seeds. Identification of genes determining flowering has important practical applications for tomato breeding. Here we demonstrate the roles of the *FANTASTIC FOUR* (*FAF*) gene family in regulating tomato flowering time. In this plant‐specific gene family, *SlFAF1/2a* shows a constitutive expression pattern during the transition of the shoot apical meristem (SAM) from vegetative to reproductive growth and significantly influences flowering time. Overexpressing *SlFAF1/2a* causes earlier flowering compared with the transformations of other genes in the *FAF* family. *SlFAF1/2c* also positively regulates tomato flowering, although to a lesser extent. The other members of the *SlFAF* gene family, *SlFAF1/2b*, *SlFAF3/4a* and *SlFAF3/4b*, are negative regulators of tomato flowering and *faf1/2b*, *faf3/4a* and *faf3/4b* single mutants all display early flowering. We generated a series of early flowering mutants using the CRISPR/Cas9 editing system, and the *faf1/2b faf3/4a faf3/4b* triple mutant flowering earliest compared with other mutants. More importantly, these mutants show no adverse effect on yield. Our results have uncovered the role of the *FAF* gene family in regulating tomato flowering time and generated early flowering germplasms for molecular breeding.

## Introduction

In plants, a pool of pluripotent cells in the growing tips forms the shoot apical meristems (SAM) and the activities of cells in the SAMs determine the shoot developmental process. During the vegetative phase, SAMs generate leaves and stems (Zhang *et al*., 2022). After floral initiation, SAMs develop into an inflorescence meristem (IM) and give rise to flowers (Benlloch *et al*., 2007; Kwiatkowska, 2008). The development of vegetative and reproductive meristems along the main axes varies in different flowering plants and the growth habit of higher plants can be divided into two categories, monopodial and sympodial (Weberling, 1992). The model species *Arabidopsis thaliana* presents a typical monopodial growth habit. The SAM undergoes vegetative growth until sensing external cues and is then transformed into an inflorescence meristem (IM), which grows continuously and produces floral meristems (FM) laterally (Andrés and Coupland, 2012; Benlloch *et al*., 2007). In this way, the main axis of Arabidopsis grows indeterminately. By contrast, the main shoot of sympodial plants terminates in flowers. Tomato plants have classical determinate inflorescences where the primary shoot meristem (PSM) switches into a FM after producing 7–12 leaves and terminates in an inflorescence. A new shoot (called sympodial shoot meristem, SYM) arises below the inflorescence on the PSM and generates three leaves before terminating in an inflorescence. Again, a new SYM develops below the inflorescence from the previous SYM and generates three leaves with a terminal inflorescence. This process occurs repeatedly so that the tomato plants display an indefinite growth habit (Park *et al*., 2011; Pnueli *et al*., 1998). Therefore, flowering process relies on the dynamic development of the SAM, and the timely switching from vegetative to reproductive growth is regulated by many internal and external factors.

The importance and influence of photoperiod on flowering were noticed and examined many years ago (Garner and Allard, 1920, 1923). Later, grafting experiments showed that, in photoperiodic plants, leaves perceive day length changes and emit a systemic hormone signal, called florigen, which moves in the phloem from leaves to the vegetative shoot apical meristem that initiates flower development (Chailakhyan, 1936). After years of dedicated research, it was demonstrated that florigen is a protein hormone encoded by the *FLOWERING LOCUS T* (*FT*) gene in Arabidopsis (An *et al*., 2004). The FT protein functions as a transcriptional cofactor that is produced in leaves after exposure to long‐days (LDs) conditions and moves to the SAM to form a complex with the basic leucine zipper domain transcription factor FD, and this complex transcriptionally activates the expression of *APETALA1* (*AP1*) and *SUPPRESSOR OF OVEREXPRESSION OF CONSTANS 1* (*SOC1*) to initiate the flowering process (Andrés and Coupland, 2012; Kobayashi and Weigel, 2007; Turck *et al*., 2008). The tomato *FT* ortholog is *SINGLE FLOWER TRUSS* (*SFT*), and *sft* mutants display several phenotypes including late‐flowering under LDs and short‐days (SDs) conditions, defects in the flower meristem identity and abnormal developments of flower organs and sympodial shoots (Molinero‐Rosales *et al*., 2004). The *sft* phenotypes can be complemented by graft‐transmissible *SFT* signals, which suggests that the florigen pathway is conserved in tomato, and *SFT* is an important regulator of flowering time, like *FT* in Arabidopsis (Lifschitz *et al*., 2006; Lifschitz and Eshed, 2006). However, the regulatory network of *SFT* shows differences compared with *FT*. In Arabidopsis, *FD* is specifically expressed in the SAM, but the tomato *FD* homologue, *SPGB* (a bZIP G‐box protein), is expressed in leaves. Therefore, unlike FT and FD, SFT may not bind with FD specifically in the SAM, which indicates that the SFT‐integrator genes and their targets may be different in tomato and Arabidopsis (Lifschitz *et al*., 2006). Thus, the model of tomato flowering regulation favours a florigen‐like model, in which the downstream pathway is relatively incomplete and requires further investigation.

The *FANTASTIC FOUR* gene family encode a class of plant‐specific protein with unknown functions, which includes four members in Arabidopsis, that is, *FAF1*, *FAF2*, *FAF3* and *FAF4*. Initially, the *FAF1* and *FAF2* genes were observed to respond strongly and rapidly to photoperiod changes (Schmid *et al*., 2003). Further investigations of the four members of the *FAF* family revealed the potential function of FAF proteins. It was shown that *FAF2* and *FAF4* are expressed in the central zone of shoot meristem that overlaps with the expression region of *WUSCHEL* (*WUS*), and the FAF2 and FAF4 proteins can arrest the expression of *WUS*. The expression of *FAF2* and *FAF4* is also under negative control by CLAVATA3 (CLV3). These results suggest that in Arabidopsis the *FAF* genes are involved in a classical CLV3‐WUS feedback loop that regulates the shoot meristem development (Wahl *et al*., 2010). The four *FAF* genes are also, however, expressed in the developing and mature vasculature (Wahl *et al*., 2010). Further, through analysing the transcriptome of Arabidopsis floral transition meristem, it was found that *FAF2* expression is significantly increased in the meristem after changing the growth conditions from SDs to LDs. The activation of *FAF2* in the floral transition meristems is dependent on the presence of *FT* and *TSF* (*TWIN SISTER OF FT*) (Torti *et al*., 2012).

*FAF* genes are also involved in other processes. The hormone cytokinin (CK) activates the growth of buds whereas auxin inhibits growth through apical dominance. Supplying buds with CK overcomes the auxin‐mediated bud inhibition. During this process, the expression levels of *FAF1*, *FAF2* and *FAF3* are significantly decreased under apical auxin and increased under CK treatment, suggesting that the *FAF1*‐*3* genes are involved in bud development (Bhargava *et al*., 2013; Muller *et al*., 2015). *FAF1* also responds to GR‐REV and KAN1‐GR induction, which may function as a meristem regulator involved in an Ad/Abaxial regulatory network (Reinhart *et al*., 2013). Therefore, the four *FAF* genes show functional divergence in Arabidopsis. Furthermore, the numbers of *FAF* genes differ between monocotyledonous and dicotyledonous species (Wahl *et al*., 2010). In tomato, the *CELL SIZE REGULATOR* (*CSR*) underlies the *fw11.3* locus, and *CSR* is mainly expressed in fruit tissues and vascular bundles during the fruit maturation process, which ultimately lead to increases in the mesocarp cell size and fruit weight (Mauxion *et al*., 2021; Mu *et al*., 2017). Phylogenetic analysis showed that the *SlCSR* gene is orthologous to *AtFAF*‐like gene, and protein sequence analysis showed that there are 13 proteins containing FAF domain in the tomato genome, three more than in Arabidopsis. It has been suggested that the expansion of the number of *FAF* gene family might only occur in the Solanaceace family (Mu *et al*., 2017). Therefore, we explored the function of the tomato FAF orthologs.

During tomato flowering, the transition meristem will eventually develop into a flower meristem, and previous studies have thoroughly characterized this process using single‐meristem transcriptomes technology (Meir *et al*., 2021). We noticed that the expression level of a *FAF* family gene was significantly downregulated in the *sft* mutant during floral transition stages (Meir *et al*., 2021). According to the phylogenic relationship in Arabidopsis, the *FAF* gene family are divided into two branches in tomato, the FAF1/FAF2 branch including *SlFAF1/2a*, *SlFAF1/2b* and *FAF1/2c* and the FAF3/FAF4 branch including *SlFAF3/4a* and *FAF3/4b*. *SlFAF1/2a* is expressed in all SAM development stages with high expression levels in floral transition meristems. Furthermore, we showed that overexpressing *SlFAF1/2a* will lead to the earliest flowering. We also characterized the function of other genes from *FAF* family, *SlFAF1/2c* is another positive regulator and *SlFAF1/2b*, *SlFAF3/4a* and *SlFAF3/4b* are three negative regulators. Based on that, we generated a series of early flowering mutants between *SlFAF1/2b*, *SlFAF3/4a* and *FAF3/4b* using the CRISPR/Ca9 editing system.

## Results

### *SlFAF1/2a* is mainly expressed during floral transition phases

In Arabidopsis, the *FAF* gene family includes four members: *FAF1*, *FAF2*, *FAF3* and *FAF4*, and *FAF1* is paralogous with *FAF2*, *FAF3* is paralogous with *FAF4* (Wahl *et al*., 2010). Phylogenetic analysis showed that there are five *FAF* genes in tomato and Solyc06g084280, Solyc06g008990 and Solyc09g065140 are homologous with *FAF1*/*FAF2* paralog, therefore, they were named as *SlFAF1/2a*, *SlFAF1/2b* and *SlFAF1/2c*, respectively. Solyc01g079740 and Solyc06g054310 are homologous with *FAF3*/*FAF4* paralog and were named *SlFAF3/4a* and *SlFAF3/4b*, respectively (Figure S1). The nomenclature of the *SlFAFs* genes follows a previous study (Mu *et al*., 2017). We compared the amino acid sequences of these five SlFAF proteins and sequence alignment results showed that in addition to the FAF conserved domain, their amino acid sequences display with many other similarities (Figure S2). These structural similarities suggested that the *SlFAF* gene family members may exhibit similar regulatory properties in tomato flowering. But there are also some differences in their amino acid sequences, so it is also possible of functional divergences between these genes.

**Figure 1:**
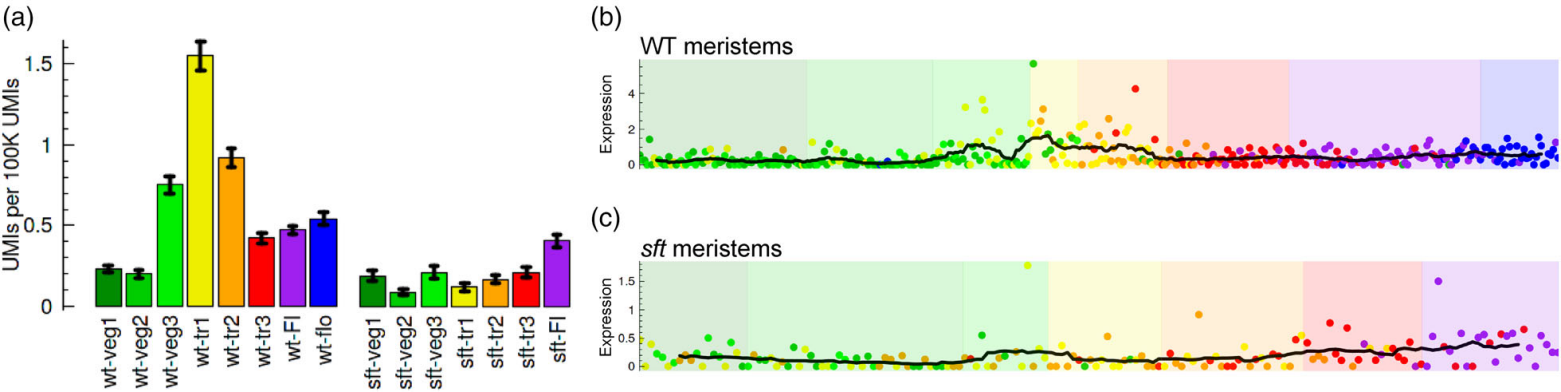
Transcriptional dynamics of *SlFAF1/2a*. (a), Expression levels of *SlFAF1/2a* in different SAM developmental phases from WT and the *sft* mutant. Vegetative phase was divided into three subphases (i.e. veg1‐3). Three transition phases: tr1, tr2 and tr3. Flower initiation phase: FI. Floral meristem formed phase: flo. Expression levels on the *y* axis were computed as the unique molecular identifiers (UMIs) per 100 000 UMIs. (b, c), Expression levels of *SlFAF1/2a* in individual meristems from WT (b) and the *sft* mutant (c). A total of 379 meristems were measured in WT and 162 meristems were measured in the *sft* mutant. The background colours represent different developmental phases, which are consistent with the colours in A. The transcriptome data and graphs were automatically produced on the website: https://tanaylab.weizmann.ac.il/SMT/.

Previous study sampled hundreds of individual tomato SAMs to perform single‐meristem transcriptome (SMT) profiling and establish a detailed dynamic transcriptome map of the floral transition process (Meir *et al*., 2021). To capture the temporal changes in genes expression levels at different developmental stages, Meir et al. ordered these individual SAMs into six phases, including vegetative phase (further divided into three subphases, i.e. veg1‐3), three transition phases (i.e. transition I, transition II and transition III), flower initiation phase and floral meristem formed phase. In the released data, we found that among these five *SlFAF* genes, only *SlFAF1/2a* is highly expressed in the SAMs at all developmental stages in a specific pattern, with the other four *SlFAF* genes showing much lower expression levels without clear expression pattern (Figure 1a, Figure S3). Thus, first we focus on the gene *SlFAF1/2a*.

Specifically, the expression level of *SlFAF1/2a* is lowest in the early vegetative phases (i.e. veg1‐2) and gradually increases in the later vegetative phase (i.e. veg3). Its highest expression level was in SAMs that were dooming (showing enlargement characteristic of floral meristem development) in the transition I phase (i.e. tr1) and subsequently decreased in the transition II phase (i.e. tr2). Following SAM bifurcation, the expression level of *SlFAF1/2a* decreased slightly in the late transition meristems (i.e. tr3 phase) and was maintained at a relatively stable level during flower initiation and formation (i.e. FI and flo phases) (Figure 1a). Intriguingly, we also noticed that in the *sft* mutant, the expression level of *SlFAF1/2a* was significantly lower in all *sft* meristems from the five developmental phases (Figure 1b, c). The most notable reduction (5.91‐fold) was in the early transition I phase in the *sft* mutant compared with the wild type. In the vegetative, transition II, transition III and floral initiation phases, *SlFAF1/2a* was also downregulated approximately 2.43‐fold, 2.32‐fold, 1.32‐fold and 1.15‐fold, respectively (Figure 1a). The high expression level of *SlFAF1/2a* in flower transition meristems indicates that this gene may be involved in the regulation of tomato flowering, and *SlFAF1/2a* may function in the downstream regulatory network of SFT.

### *SlFAF1/2a* positively regulates tomato flowering time

**Figure 2:**
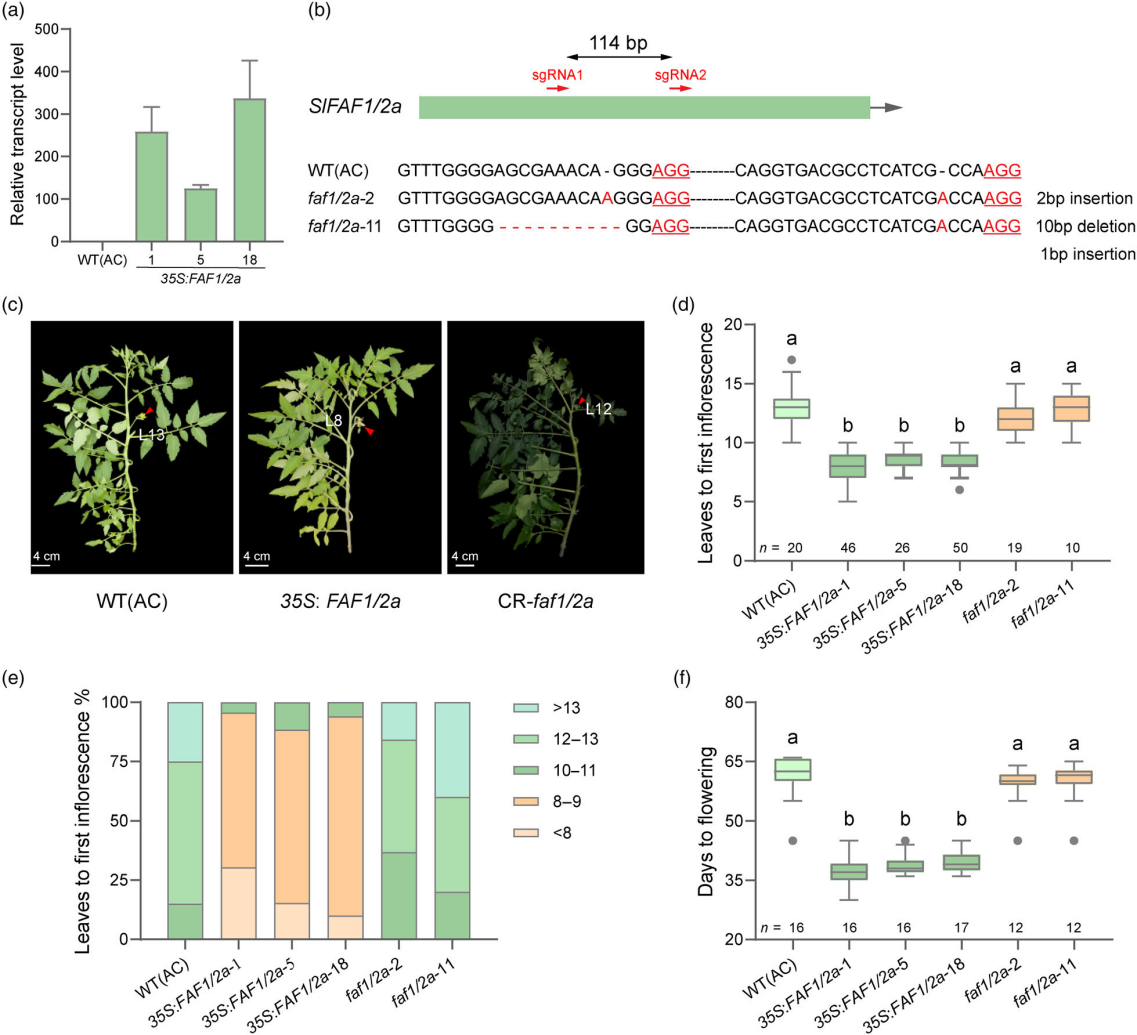
Functional characterizations of *SlFAF1/2a*. (a) Relative transcript levels of *SlFAF1/2a* in WT (AC) and the *SlFAF1/2a*‐overexpressing lines. Three biological replicates were analysed for each line. Error bars indicate SE. (b) Generation of *faf1/2a* mutants by CRISPR/Cas9. The sgRNAs sequences of *SlFAF1/2a* in the WT (AC) and in the *faf1/2a*‐2, *faf1/2a*‐11 mutants are shown. (c) Plants from WT (AC), *SlFAF1/2a*‐overexpressing lines and CR‐*faf1/2a* mutants. The first inflorescences are indicated with red arrows and the number of leaves before the first inflorescences are indicated on the Figures. (d) Distribution of number of leaves before the first inflorescences in WT (AC), *SlFAF1/2a*‐overexpressing lines and CR‐*faf1/2a* mutants. (e) Percentages of number of leaves before the first inflorescences of WT (AC), *SlFAF1/2a*‐overexpressing lines and CR‐*faf1/2a* mutants. (f) Distribution of days to flowering in WT (AC), *SlFAF1/2a*‐overexpressing lines and CR‐*faf1/2a* mutants. *n*, number of plants investigated. All distributions are shown as box plots of the range of percentiles from the total data, as determined using Tukey's method. Central line, median; whiskers, interquartile range; outer dots, outliers. Different letters indicate significant differences (*P* < 0.0001, one‐way ANOVA with Tukey's post hoc test).

To investigate the function of *SlFAF1/2a* in regulating tomato flowering time, we overexpressed it in the cultivated accession Ailsa Craig (AC), and obtained three independent overexpressing transgenic lines, *35S*: *FAF1/2a*‐1, *35S*: *FAF1/2a*‐5 and *35S*: *FAF1/2a*‐18 (Figure 2a). We also mutagenized *SlFAF1/2a* in the wild‐type (WT) using CRISPR/Cas9‐mediated genome editing (Figure 2b). We sequenced the resulting *faf1/2a* alleles in independent *faf1/2a* mutants and selected two independent homozygous lines (*faf1/2a*‐2 and *faf1/2a*‐11) (Figure 2b). The transgenic plants and WT (AC) plants were grown under the same environmental conditions. The number of leaves formed before the first inflorescence developed and the days to flowering by each plant were counted.

All WT (AC) plants produced more than 10 leaves before the first inflorescence, with a majority (85%) of plants producing more than 12 leaves before the first inflorescence (Figure 2c–e). Remarkably, the number of leaves formed before the first inflorescence was significantly reduced in the *SlFAF1/2a*‐overexpressing lines (Figure 2d). For all overexpressing lines, the maximum number of leaves before the first inflorescence was 10 (Figure 2d). The majority, 95.65%, 88.46% and 94% in *35S*: *FAF1/2a*‐1, *35S*: *FAF1/2a*‐5 and *35S*: *FAF1/2a*‐18 lines, respectively, produced fewer than nine leaves before the first inflorescence (Figure 2e). In addition to the effects of *SlFAF1/2a* on the number of leaves produced before the first inflorescence, the days to flowering were also significantly reduced in the *SlFAF1/2a*‐overexpressing lines. The average number of days before flowering was 61 in the WT (AC) plants, which was reduced to 37.19, 38.75 and 39.88 in the three *SlFAF1/2a*‐overexpressing lines, respectively (Figure 2f). Therefore, these results show that the increased expression level of *SlFAF1/2a* resulted in marked earlier flowering in tomato. When we investigated the CR‐*faf1/2a* mutants, there are no significant differences in the number of leaves produced before the first inflorescence and the number of days before flowering compared with WT (AC) plants (Figure 2c–f). Thus, loss of SlFAF1/2a function did not alter the flowering time significantly. Taken together, these results demonstrated that *SlFAF1/2a* positively regulate tomato flowering time.

### Different members of the *SlFAF* gene family have contrasting effects on tomato flowering

To explore the function of other *SlFAF* genes, we overexpressed *SlFAF1/2b*, *SlFAF1/2c*, *SlFAF3/4a* and *SlFAF3/4b* separately in the AC background and obtained three independent overexpressing lines for each gene (Figure S4A–D). We also mutagenized *SlFAF1/2b*, *SlFAF1/2c*, *SlFAF3/4a* and *SlFAF3/4b* in the same background using CRISPR/Csa9 technology (Figure S4E–H). We sequenced the resulting *faf1/2b*, *faf1/2c*, *faf3/4a* and *faf3/4b* alleles in independent *faf1/2b*, *faf1/2c*, *faf3/4a* and *faf3/4b* mutants, respectively, and selected two homozygous mutants for each gene (Figure S4E–H).

**Figure 3:**
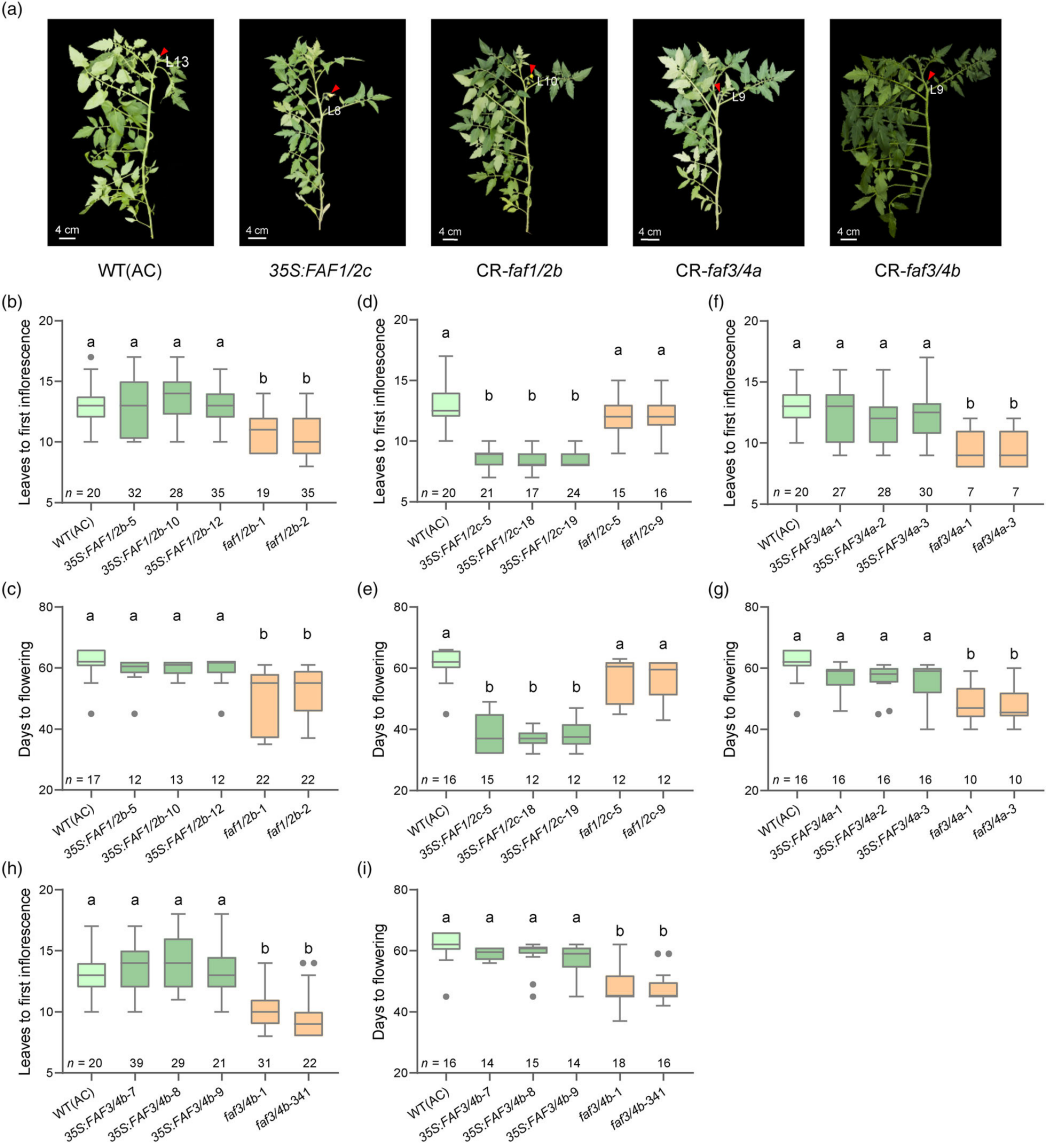
Functional characterizations of *SlFAF1/2b*, *SlFAF1/2c*, *SlFAF3/4a* and *SlFAF3/4b*. (a) Plants from WT (AC), *35S*: *SlFAF1/2c*, CR‐*faf1/2b*, *faf3/4a* and *faf3/4b* mutants. The first inflorescences are indicated with red arrows and the number of leaves before the first inflorescences are indicated on the Figures. (b, d, f and h) Distribution of number of leaves before the first inflorescences in WT (AC) and *SlFAF1/2b* (b), *SlFAF1/2c* (d), *SlFAF3/4a* (f), *SlFAF3/4b* (h) overexpressing lines and mutant lines. (c, e, g and i) Distribution of days to flowering in WT (AC) and *SlFAF1/2b* (c), *SlFAF1/2c* (e), *SlFAF3/4a* (g), *SlFAF3/4b* (i) overexpressing lines and mutant lines. *n*, number of plants investigated. All distributions are shown as box plots of the range of percentiles from the total data, as determined using Tukey's method. Different letters indicate significant differences (*P* < 0.05, one‐way ANOVA with Tukey's post hoc test).

In the *FAF1*/*FAF2* evolutionary branch, the regulatory effect of *SlFAF1/2c* is similar to *SlFAF1/2a*. Overexpressing *SlFAF1/2c* leads to early flowering (Figure 3a). The number of leaves before the first inflorescence of *SlFAF1/2c*‐overexpressing lines was between 7 and 10, which is significantly reduced compared with WT (AC) (Figure 3d). The number of days before flowering was also significantly reduced to 37 days in the *SlFAF1/2c*‐overexpressing lines compared with 61 days in WT (AC) (Figure 3e). And no differences were observed in the flowering time comparing CR‐*faf1/2c* mutants with WT (AC) (Figure 3d,e). In contrast, overexpressing *SlFAF1/2b* did not alter the flowering time (Figure 3b,c). In the CR‐*faf1/2b* mutants, however, the number of leaves before first inflorescence developed was lower to 10 compared to 13 leaves in WT (AC), which is significantly reduced (Figure 3a,b). The number of days before flowering also significantly reduced to 51 days in the *faf1/2b* mutants (Figure 3c), which represents a significant acceleration of the flowering process compared with WT (AC). Therefore, the *SlFAF1/2a* and *SlFAF1/2c* display similar regulations on tomato flowering, and their action is different from *SlFAF1/2b*.

In the *FAF3*/*FAF4* branch, the transgenic lines of *SlFAF3/4a* and *SlFAF3/4b* exhibited similar phenotypic changes as the *SlFAF1/2b* transgenic lines. A majority of plants from *SlFAF3/4a‐*overexpressing lines produced over 12 leaves before the first inflorescence, which is similar to WT (AC) (Figure 3f), and the number of days before flowering are also similar to WT (AC) (Figure 3g). But in the CR‐*faf3/4a* mutants, the number of leaves before the first inflorescence developed was lower to 9 compared to 13 leaves in WT (AC), which is significantly reduced (Figure 3a,f). The number of days before flowering also significantly reduced to 48 days in the *faf3/4a* mutants (Figure 3g). Similarly, the flowering time of *SlFAF3/4b*‐overexpression lines is not altered (Figure 3h,i). In the CR‐*faf3/4b* mutants, the number of leaves before first inflorescence developed was lower to 9, which is significantly reduced compared with WT (AC) (Figure 3a,h). The number of days before flowering also significantly reduced to 47 days in the *faf3/4a* mutants (Figure 3i). Thus, the loss of the function of *SlFAF3/4a* and *SlFAF3/4b* leads to early flowering. Taken together, these transformation experiments demonstrated that in the *SlFAF* gene family, the *SlFAF1/2a* and *SlFAF1/2b* are the positive regulators and *SlFAF1/2b*, *SlFAF3/4a*, *SlFAF3/4b* are the negative regulators for tomato flowering time.

### Additive effects among the negative regulators in *SlFAF* gene family on tomato flowering

Knocking out *SlFAF1/2b*, *SlFAF3/4a* and *SlFAF3/4b* caused early flowering (Figure 3b,c,f–i) and the average number of leaves before the first inflorescences were 10, 9 and 9 in CR‐*faf1/2b*, CR‐*faf3/4a* and CR‐*faf3/4b* lines, respectively, which was 3–4 leaves earlier than in WT (AC) that produces an average of 13 leaves before the first inflorescence develops. To explore whether knocking out these three genes simultaneously would result in further early flowering, we took advantage of the CRISPR/Cas9 multiplex editing capability (Xie *et al*., 2015) to generate the double mutants *faf1/2b faf3/4b*, *faf3/4a faf3/4b* (Figure S5A,B) and the triple mutants *faf1/2b faf3/4a faf3/4b* (Figure S5C), respectively. These mutants were generated in the AC background. For each mutant combination, we selected two homozygous lines separately (Figure S5).

**Figure 4:**
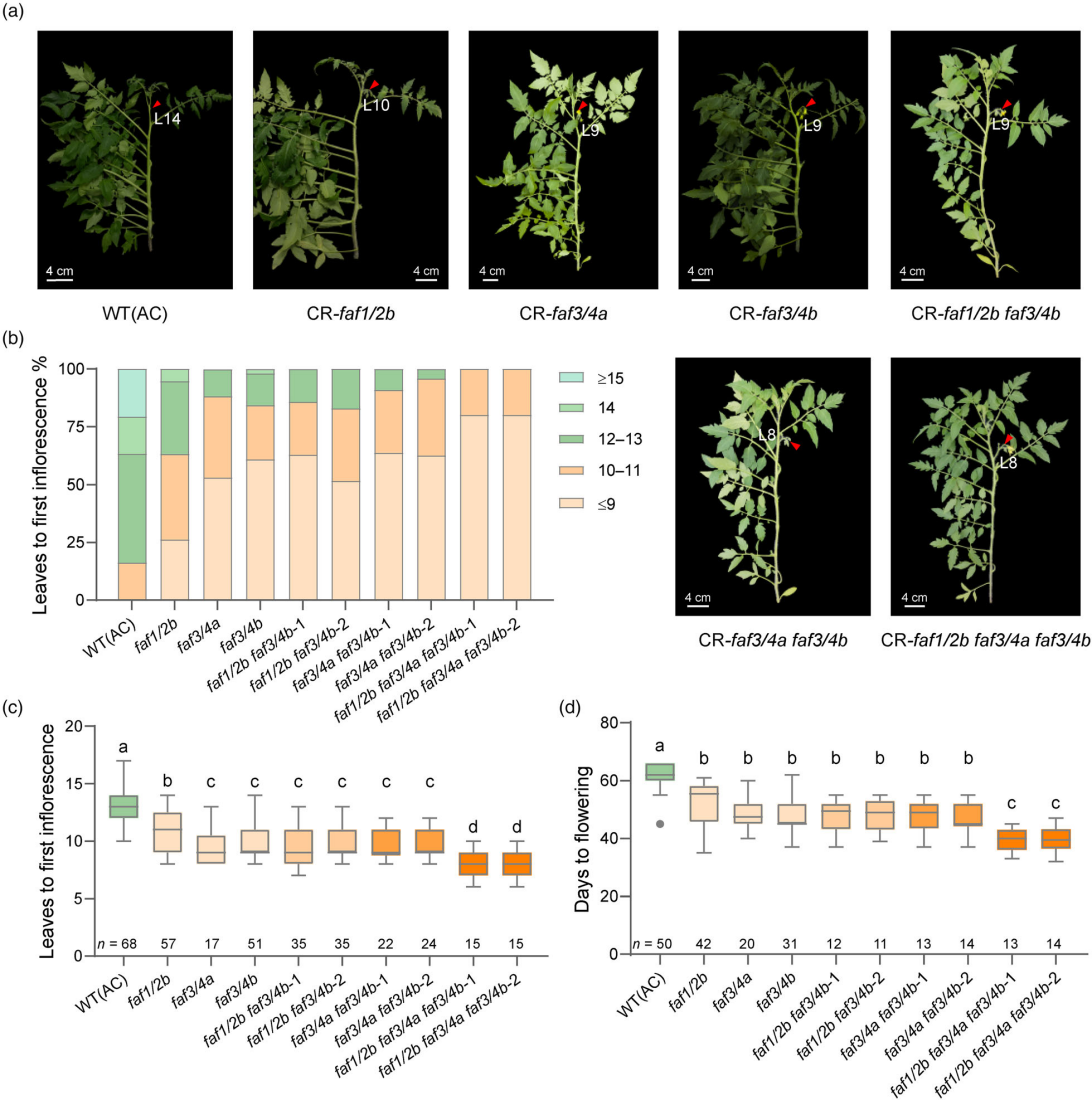
Additive effects among the negative regulators in the *SlFAF* gene family. (a) Plants from WT (AC), CR‐*faf1/2b*, *faf3/4a* and *faf3/4b* single mutants, *faf1/2b faf3/4b*, *faf3/4a faf3/4b* double mutants and *faf1/2b faf3/4a faf3/4b* triple mutants. The first inflorescences are indicated with red arrows and the number of leaves before the first inflorescences are indicated on the Figures. (b) Percentages of number of leaves before the first inflorescences in WT (AC) and different single, double and triple mutants. (c) Distribution of number of leaves before the first inflorescences in WT (AC) and different single, double and triple mutants. (d) Distribution of days to flowering in WT (AC) and different single, double and triple mutants. *n*, number of plants investigated. All distributions are shown as box plots of the range of percentiles from the total data, as determined using Tukey's method. Different letters indicate significant differences (*P* < 0.05, one‐way ANOVA with Tukey's post hoc test).

The number of leaves produced before the first inflorescence and the number of days before flowering in WT (AC) and different mutant lines were quantified. In WT (AC), approximately 83.82% of plants formed more than 12 leaves before flowering and the average number is 13 leaves, the average number of days before flowering is 62 days (Figure 4b–d). As described above, the single mutants of *faf1/2b*, *faf3/4a* and *faf3/4b* cause early flowering compared with WT (AC) (Figure 3). Among these three single mutants, in the *faf1/2b* mutant, the reduction in number of leaves before flowering is to a lesser degree compared with *faf3/4a* and *faf3/4b* single mutant, the number of days before flowering is not significantly different from *faf3/4a* and *faf3/4b* single mutant (Figure 4c,d). When we combined the *faf1/2b* with *faf3/4b*, and the *faf1/2b faf3/4b* double mutants exhibited similar flowering time distributions compared with *faf3/4b* single mutants (Figure 4b–d). Thus, the *faf1/2b* did not show an additive effect on *faf3/4b*. The combination between *faf3/4a* and *faf3/4b* mutations also shows no additional effect on flowering time compared with the single mutant (Figure 4b–d). Further, we generated the *faf1/2b faf3/4a faf3/4b* triple mutants. In the triple mutants, the average number of leaves before first inflorescence was 8 and the average number of days before flowering was 39 days, which is significantly reduced compared with WT (AC), the single and double mutants (Figure 4c,d). Thus, the triple mutants exhibit the most pronounced early flowering phenotype. Taken together, we showed that in the *FAF* gene family, the combined mutations between the single mutants of the negative regulators will cause the earliest flowering.

### No adverse effects on plant development and yield in early flowering *slfafs* mutants

To explore whether the *SlFAF* gene family show other effects on plants following the alteration of flowering time, we investigated the plant growth, flower and inflorescence development, fruit set rates, fruit development and plant yield of the *SlFAFs* transgenic lines and WT (AC).

**Figure 5:**
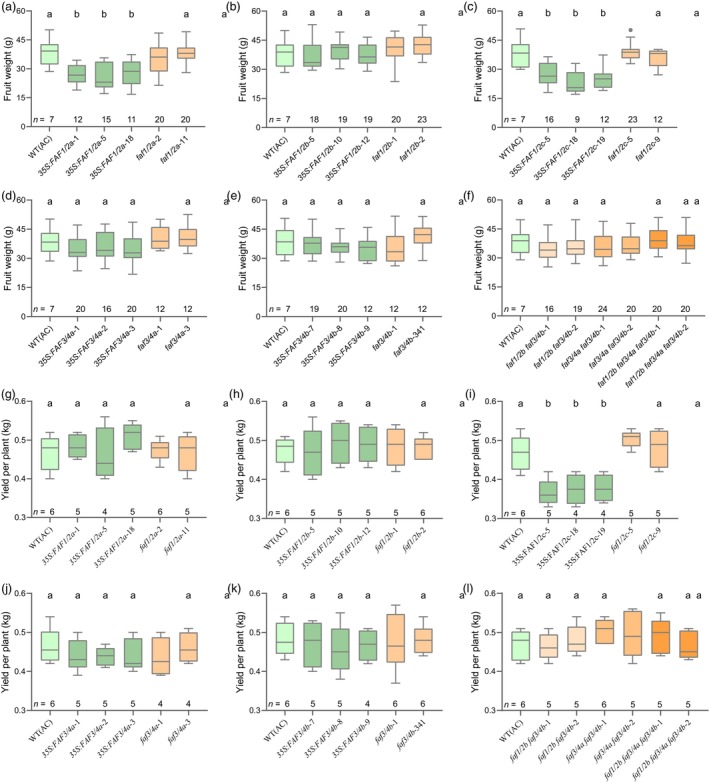
Fruit weight and yield of *SlFAFs* overexpression and mutant lines. (a–f) Distribution of single fruit weight (g) in WT (AC) and *SlFAF1/2a* (a), *SlFAF1/2b* (b), *SlFAF1/2c* (c), *SlFAF3/4a* (d), *SlFAF3/4b* (e) overexpressing lines and single mutant lines, double and triple mutant lines (f). *n*, number of fruits investigated. (g–l) Distribution of fruit yield in WT (AC) and *SlFAF1/2a* (g), *SlFAF1/2b* (h), *SlFAF1/2c* (i), *SlFAF3/4a* (j), *SlFAF3/4b* (k) overexpressing lines and single mutant lines, double and triple mutant lines (l). Yield from each plant were counted at 130 days. *n*, number of plants investigated.

**Figure 6:**
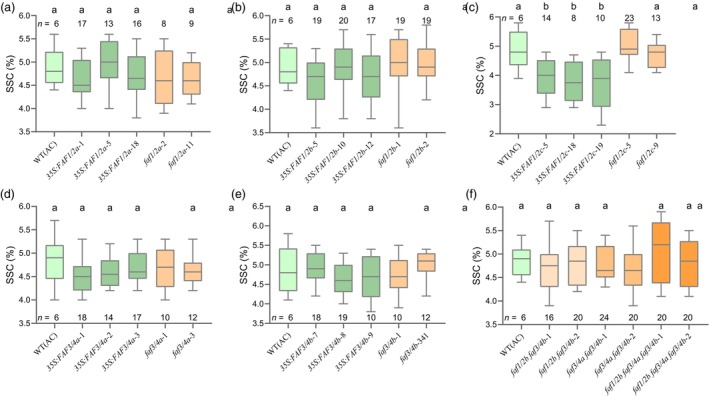
Soluble solids content of *SlFAFs* overexpression and mutant lines. (a–f) Distribution of soluble solids content (%) in WT (AC) and *SlFAF1/2a* (a), *SlFAF1/2b* (b), *SlFAF1/2c* (c), *SlFAF3/4a* (d), *SlFAF3/4b* (e) overexpressing lines and single mutant lines, double and triple mutant lines (f). *n*, number of fruits investigated.

At 110 days, plants from early flowering *SlFAFs* transgenic lines grow similarly with WT (AC) plants without defections (Figure S6). For the flower development, there are no significant differences in their flower size and structure comparing different transgenic lines with the WT (AC) (Figure S7A), and the flower number from each inflorescence of the transgenic lines is similar to WT (AC), which is mainly distributed between 8 and 10 (Figure S7B–G). The inflorescences from the transgenic lines still develop one branch, which are same as the WT (AC) (Figure S7H). Therefore, the flower and inflorescence development remain unaffected in the *SlFAF* transgenic lines. In addition, we investigated the fruit set rates in the transgenic lines, which are similar to the WT (AC) (Figure S8). The fruits from *SlFAF1/2a* and *SlFAF1/2c*‐overexpression lines are smaller than the WT (AC) (Figure 5a,c). However, the total yield from *SlFAF1/2a*‐overexpression plants is not changed, while they are reduced in the *SlFAF1/2c*‐overexpression plants (Figure 5g,i). For other transgenic lines, no differences were observed in the fruit size or plant yield comparing with WT (AC) (Figure 5). Besides that, the soluble solids content of fruits from *SlFAF1/2c*‐overexpression lines also reduced relative to WT (AC) (Figure 6c), but not in other transgenic lines (Figure 6a,b,d–f). Regarding fruit shape, fruits from *SlFAF3/4a* and *SlFAF3/4b*‐overexpression lines are longer than the WT (AC), but not in other transgenic lines (Figure S9). Taken together, the early flowering *SlFAF1/2a*‐overexpression lines and the CR‐*faf1/2b*, *faf3/4a*, *faf3/4b*, *faf1/2b faf3/4b*, *faf3/4a faf3/4b*, *faf1/2b faf3/4a faf3/4b* mutants have no side effect on plant yield.

## Discussion

In this study, we characterized the function of the *FAF* gene family in tomato and showed that they have different regulatory effects on tomato flowering time. Increasing the expression level of *SlFAF1/2a* had the most significant effect on tomato flowering. Most plants from the *SlFAF1/2a*‐overexpressing lines produced less than 8 leaves before the first inflorescence, and it took about 30 days before flowering, which is drastically shortened compared with WT (AC), which took on average 61 days and 13 leaves before flowering (Figure 2). The other positive regulator is *SlFAF1/2c* and its effect on early flowering was also significant, albeit to a lesser extent than *SlFAF1/2a* (Figure 3d,e). Mutations in the remaining three genes, *SlFAF1/2b*, *SlFAF3/4a* and *SlFAF3/4b*, caused early flowering (Figure 3a). *SlFAF1/2b* forms a branch with *SlFAF1/2a* and *SlFAF1/2c*, but display the opposite regulation on flowering. This may be caused by the different expression pattern between *SlFAF1/2b* and *SlFAF1/2a* or *SlFAF1/2c* (Figure S3). *SlFAF3/4a* and *SlFAF3/4b* formed the other branch in *SlFAF* gene family (Figure S1). Both of them are negative regulators of tomato flowering (Figure 3f–i).

In tomato, most natural mutants related to flowering time show delayed flowering, such as *falsiflora* (*fa*), *compound inflorescence* (*s*), *jointless* (*j*), *single flower truss* (*sft*) and *uniflora* (*uf*) (Dielen *et al*., 2004; Lifschitz *et al*., 2006; Lippman *et al*., 2008; Molinero‐Rosales *et al*., 1999; Szymkowiak and Irish, 2006). In contrast, the *terminating flower* (*tmf*) is the only identified early flowering mutant in tomato (MacAlister *et al*., 2012). Early flowering usually means early yield, which is important for tomato production. To acquire early flowering germplasms, previous studies used the CRISPR/Cas9 system to engineer mutations in the flowering repressor *SELF‐PRUNING 5G* (*SP5G*) (Soyk *et al*., 2017). These *sp5g* mutants displayed rapid flowering and the quick flower production subsequently translated to early yield (Soyk *et al*., 2017). We showed that individually mutagenizing *SlFAF1/2b*, *SlFAF3/4a* and *SlFAF3/4b* also leads to early flowering. To explore the genetic effects on tomato flowering between these three genes, we generated the *faf1/2b faf3/4b*, *faf3/4a faf3/4b* and *faf1/2a faf3/4a faf3/4b* multiple gene mutants. The double mutants did not show an addictive effect compared with the single mutants (Figure 4c,d). Simultaneous mutations in *SlFAF1/2b*, *SlFAF3/4a* and *SlFAF3/4b* significantly accelerated flowering time compared with the single and double mutants, which suggests that there are possible additive effects underlying the action of these three genes (Figure 4b–d). Taken together, we created a series of tomato mutants with different degrees of early flowering, which assist breeders to select germplasms of different flowering times.

In the early flowering *faf1/2b*, *faf3/4a*, *faf3/4b* single mutants and the *faf1/2b faf3/4b*, *faf3/4a faf3/4b*, *faf1/2a faf3/4a faf3/4b* multiple gene mutants, we found that their plant growths were unaffected and the fruits mature earlier comparing with WT (AC) owing to early flowering in the mutants (Figure S6). In addition, the flowers, inflorescences and fruit development from those mutants were also unaffected (Figures S7, S9F and Figure 6f). More importantly, since the fruit set rates and fruit weight still were similar to WT (AC), which the plant yield was not affected either (Figure S8F, Figure 5f,l). Taken together, we show that mutations in *SlFAF* genes cause earlier flowering without significant adverse effects on plant development and yield.

In summary, we discovered a gene belonging to *FAF* gene family (i.e. *SlFAF1/2a*) that is highly expressed during floral transition stages, and its expression level is influenced by SFT. Overexpressing *SlFAF1/2a* caused early flowering, knocking out this gene had no influence on flowering time, compared to WT (AC). Characterization of the remaining genes from this family demonstrated that *SlFAF1/2a* and *SlFAF1/2c* promote early flowering, whereas *SlFAF1/2b*, *SlFAF3/4a* and *SlFAF3/4b* are flowering repressors. Knocking out either *SlFAF1/2b*, *SlFAF3/4a* or *SlFAF3/4b* caused early flowering, and simultaneously mutagenizing these three genes resulted in the earliest flowering time without adverse effects on yield compared with the other single and double mutants.

## Methods

### Plant materials and growth conditions

The cultivated tomato, *Solanum lycopersicum* cv. Ailsa Craig (AC), was selected for *Agrobacterium tumefaciens* (strain C58)‐mediated transformation experiments (Ouyang *et al*., 2005). The transgenic plants from T_2_ generations were investigated. All plants were grown under the same environmental conditions: photoperiod consisting of 16 h of light and 8 h of darkness, temperature at 25 ± 2 °C, and a relative humidity of 70%.

### Plants phenotyping

Individual plants from the transgenic lines and wild‐type (AC) were used to count the number of leaves formed before the first inflorescence developed. Days to flowering were counted from the first day of sowing seeds. At least seven plants were investigated for each line. For flower and inflorescence development, they were investigated using the second or third inflorescence from each transgenic line and WT (AC). At least six inflorescences were investigated for each line. Regarding fruit development, at least six fruits were investigated for fruit size, fruit shape and soluble solids content (SSC). SSC was quantified as Brix from red‐ripe fruits using a digital refractometer (PAL‐BX|ACID3). Fruit set rates were counted from the second or third inflorescence of each line. At least six inflorescences were investigated. Plant yields were counted using at least five plants for each line. For each phenotype, the exact investigated number was presented in corresponding Figures.

### Phylogenetic analysis

Phylogenetic relationships between FAFs proteins in *S. lycopersicum* and *Arabidopsis thaliana* were inferred using the neighbour‐joining method. The percentage of replicate trees in which the associated taxa clustered together in the bootstrap test (2000 replicates) are shown next to the branches. The full‐length amino acid sequences of SlFAFs paralogous and orthologous genes were downloaded from EnsemblPlants and aligned using Clustal W2. Evolutionary analyses were conducted in MEGA7 (Kumar *et al*., 2016).

### Expression pattern analysis of *SlFAFs* in SAM

The expression levels of *SlFAFs* genes in vegetative meristems (i.e. veg1‐3), transition meristem (i.e. tr1‐3) and floral initiation meristems (i.e. FI) were downloaded from the tomato Single‐Meristem‐Transcriptome (SMT) database v3.2 (https://tanaylab.weizmann.ac.il/SMT/). The relative histograms were automatically generated on this website. These data were released from previous analysis of single‐meristem transcriptomes (Meir *et al*., 2021).

### Generation of transgenic tomato

To generate the overexpression constructs, the ORFs of *SlFAF1/2a*, *SlFAF1/2b*, *SlFAF1/2c*, *SlFAF3/4a* and *SlFAF3/4b* were amplified from AC genomic DNA and individually cloned downstream of the cauliflower mosaic virus (CaMV) 35S promoter in pHELLSGATE8 to yield the *35Spro*: *FAF1/2a*, *35Spro*: *FAF1/2b*, *35Spro*: *FAF1/2c*, *35Spro*: *FAF3/4a* and *35Spro*: *FAF3/4b* construct. The tomato accession AC was transformed with one of each of these five constructs. Positive transgenic plants were identified using a CaMV 35S promoter primer and a gene‐specific primer. After qPT‐PCR analysis, three independent homozygous transgenic lines expressing increased levels of *FAF1/2a*, *FAF1/2b*, *FAF1/2c*, *FAF3/4a* and *FAF3/4b* were selected for phenotyping.

The CRISPR‐Cas9 binary vector pTX was used to introduce mutations in the genomic region of *FAF1/2a*, *FAF1/2b*, *FAF1/2c*, *FAF3/4a* and *FAF3/4b* using the simple guide RNAs (sgRNA). The *faf1/2a*, *faf1/2b*, *faf1/2c*, *faf3/4a* and *faf3/4b* mutants were generated in the AC background by targeting two sites in their exons, that is, sgRNA1 and sgRNA2 on *FAF1/2a*, sgRNA3 and sgRNA4 on *FAF1/2b*, sgRNA5 and sgRNA6 on *FAF1/2c*, sgRNA7 and sgRNA8 on *FAF3/4a*, and sgRNA9 and sgRNA10 on *FAF3/4b* (Figure S4). The sgRNAs were designed at CRISPR‐P v2.0 (http://crispr.hzau.edu.cn/CRISPR2/) and the sequence of these sgRNAs is listed in Table S1. We sequenced the genomic region of each gene and detected the presence of vector, and selected two homozygous mutant lines lacking Cas9 protein for each of the five *FAF* genes.

The *faf3/4a faf3/4b* double mutants and the *faf1/2b faf3/4a faf3/4b* triple mutants were generated using the CRISPR‐Cas9 system. Four sgRNAs for *FAF3/4a* (i.e. sgRNA8 and sgRNA7) and *FAF3/4b* (i.e. sgRNA9 and sgRNA10) and six sgRNAs for *FAF1/2b* (i.e. sgRNA3 and sgRNA4), *FAF3/4a* (i.e. sgRNA8 and sgRNA7) and *FAF3/4b* (i.e. sgRNA9 and sgRNA10) were concatenated into pTX vector (Figure S5). These two recombinant pTX plasmids were constructed following the previously described protocol (Xie *et al*., 2015) and were individually transformed into AC. After PCR genotyping, we obtained two *faf3/4a faf3/4b* double mutant lines and two *faf1/2b faf3/4a faf3/4b* triple mutant lines without Cas9 protein.

### Quantitative real‐time PCR analysis

To detect the expression levels of *FAF* genes, total RNA from leaves of *FAFs*‐overexpression lines was extracted using TRIzol reagent (Aidlab, China). AHiScript II 1st Strand cDNA Synthesis Kit (+gDNA wiper) (Vazyme, China) was used to synthesize the first‐strand cDNA according to the manufacturer's protocol. The relative transcript levels of *FAFs* genes were determined by quantitative real‐time PCR (qRT‐PCR) on a QuantStudio™ 6 Flex System (ABI, USA). Solyc11g008430 (Q‐actin) was used as an internal control. Primer sequences are listed in Table S1.

### Statistical analysis

GraphPad Prism version 8.3.0 (GraphPad Software, Inc., La Jolla, CA: http://www.graphpad.com/) was used to perform the statistical analysis. Statistically significant differences between groups were determined using One‐way ANOVA with Tukey's post hoc test.

### Accession numbers

The gene sequences of tomato were downloaded from the Sol Genomics Database (https://solgenomics.net/) with the following accession numbers: *SlFAF1/2a* (Solyc06g084280), *SlFAF1/2b* (Solyc06g008990), *SlFAF1/2c* (Solyc09g065140), *SlFAF3/4a* (Solyc01g079740), *SlFAF3/4b* (Solyc06g054310). The *AtFAF1‐4* gene accession numbers: *AtFAF1* (AT4G02810), *AtFAF2* (AT1G03170), *AtFAF3* (AT5G19260), *AtFAF4* (AT3G06020).

## Conflict of interests

The authors declare no conflicts of interest.

## Author contributions

L.S., J.S., Y.Z. and Z.Y. designed the experiments; L.S. performed experiments and wrote the manuscript; J.S., J.T., Y.W., X.Z., P.G., H.D. F.L. and W.G. helped perform some of the experiments; D.G., Z.Y. and Y.Z. supervised the project and revised the manuscript.

## Supporting information


**Figure S1** Phylogenetic analysis of *SlFAFs* and *AtFAFs*.
**Figure S2** Sequences alignment of SlFAF proteins.
**Figure S3** Transcriptional dynamics of *SlFAF1/2b*, *SlFAF1/2c*, *SlFAF3/4a* and *SlFAF3/4b*.
**Figure S4** Generations of *SlFAF1/2b*, *SlFAF1/2c*, *SlFAF3/4a* and *SlFAF3/4b* transgenic lines.
**Figure S5** Generations of double and triple *SlFAF* mutants using CRISPR/Cas9 system.
**Figure S6** Plants growth of early flowering *SlFAFs* overexpression and mutant lines.
**Figure S7** Flower and inflorescence development of *SlFAFs* overexpression and mutant lines.
**Figure S8** Fruit set rates of *SlFAFs* overexpression and mutant lines.
**Figure S9** Fruit shape of *SlFAFs* overexpression and mutant lines.
**Table S1** List of primers used in this study.
