# Supplementary material for: CRISPR/Cas9‐mediated mutations of FANTASTIC FOUR gene family for creating early flowering mutants in tomato
Source: Plant Biotechnol J. 2023 Nov 9;22(3):774–84. doi: 10.1111/pbi.14223 (PMC10893942; doi:10.1111/pbi.14223)
Supplement: Supplementary file 1 — Figure S1 Phylogenetic analysis of SlFAFs and AtFAFs. Figure S2 Sequences alignment of SlFAF proteins. Figure S3 Transcriptional dynamics of SlFAF1/2b, SlFAF1/2c, SlFAF3/4a and SlFAF3/4b. Figure S4 Generations of SlFAF1/2b, SlFAF1/2c, SlFAF3/4a and SlFAF3/4b transgenic lines. Figure S5 Generations of double and triple SlFAF mutants using CRISPR/Cas9 system. Figure S6 Plants growth of early flowering SlFAFs overexpression and mutant lines. Figure S7 Flower and inflorescence development of SlFAFs overexpression and mutant lines. Figure S8 Fruit set rates of SlFAFs overexpression and mutant lines. Figure S9 Fruit shape of SlFAFs overexpression and mutant lines. Table S1 List of primers used in this study. [file PBI-22-774-s001.zip › Supplemental Figures.pdf]

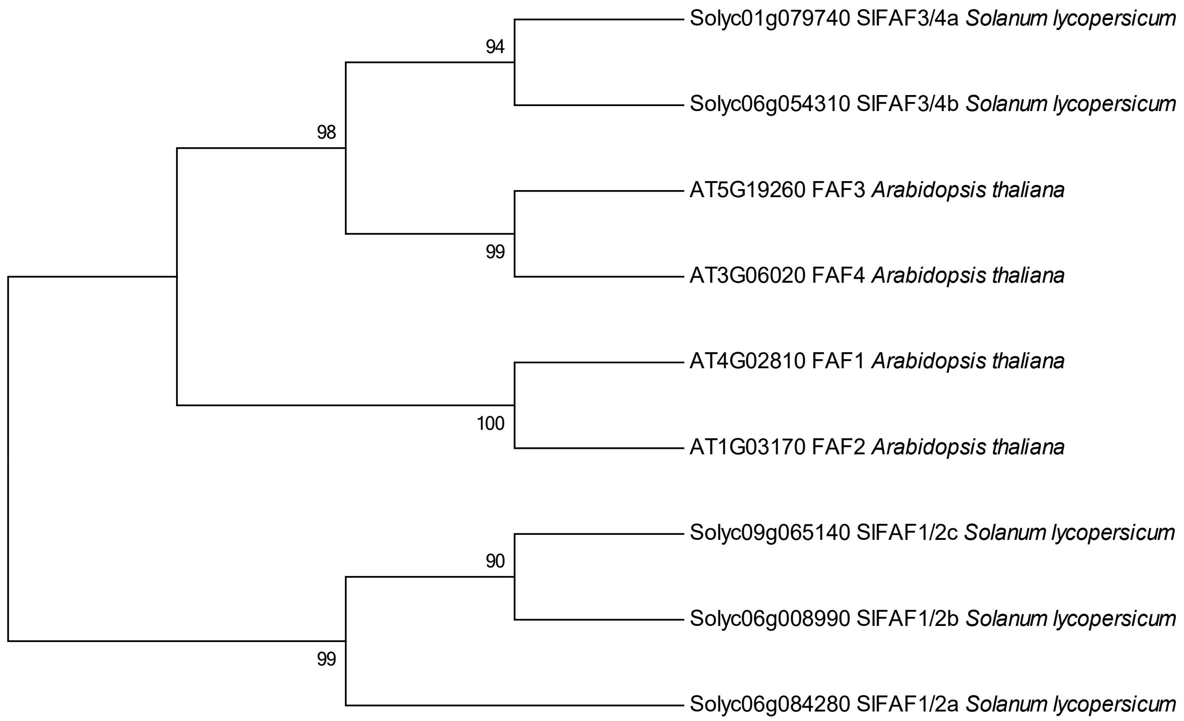

Supplemental Figure 1. Phylogenetic analysis of *SlFAFs* and *AtFAFs*. Phylogenetic tree of FAF proteins in *Solanum lycopersicum* and *Arabidopsis thaliana*. The full-length amino acid sequences of FAF paralogous and orthologous genes were downloaded from EnsemblPlants and aligned using Clustal W2. The phylogenetic tree was constructed using the neighbor-joining algorithm in MEGA 7.

```
Consensus  --------------------------------------------IN-----------N---GGWSSIQ-L-NP----------------Y
SlFAF1/2a  ----------------------------------------MDVVIN---------KVAEESGGWWSL-ILCNP-----------KREDYG 29
SlFAF1/2b  --------------------------------------------MS---------QNLEDVNSWSFIQILNNPSTYHHQIDYKKDDNDVY 37
SlFAF1/2c  MTSTSTLYQGLQSCLEPKMAPPRPNFSQPITLPQKSKICQHEDEIE---------QENDNVGGLSFIQALANPCQYSKKGD---EKDEIY 78
SlFAF3/4a  --MSTIVYQDILQ--PSQIIETT---TLKLKIPSSIEFGNVKEKCNYQVTTKKSCTTNLDCGGWSSLQNLSNIS----------KESGIY 73
SlFAF3/4b  --MSTIVYQGYQSYSDSNIIETT---TLKLKVGTPT---TIVENIN---------TSNQKC--WSSIQNLSSI-NYSN------EMEKSY 64

Consensus  -HPL-KL--------S-KSL-MCTESLGSETG-D---E-I-----------------------------S--R---K--KR--------F
SlFAF1/2a  EKQVTKL--------SIKSLEMCTESLGSETGRE-SSESIENNN--SKIVKK-----------------HTTTSSNKML--VAH-----F 84
SlFAF1/2b  IHPSTKLSSLVM---STKSLEMCTESLGNETGCY-FNESMEERDI-ARSVQR-----------------SKCREFKKRIKRTMN-----F 100
SlFAF1/2c  VHPLVKLSSL-----NTNSLNMCTESLGSETGSD-ISENIDEKETIFHGAQR-----------------SKCREFAKKIKRVAS-----Y 140
SlFAF3/4a  VHPLSKKCSYSSSRLSEKSLQLCTESLGSETGTDIIDANIFSFSSSYSRIEKSPSSTTAINDDYTMTSSSSSRNYRGNSKRSNSSVNKKF 163
SlFAF3/4b  IHRL-----------NEHSLALCTENLGSETGTD-QNIDNNNFSISSRELKT-----------------EQSNYSKVTMSYKNTQTSRKL 125
```

FAF superfamily

```
Consensus  PPPLTSI-G---VQ-RPHREGGRL-LKA--------YF-AER-NGRL-LSF---------IDE---------------------------
SlFAF1/2a  PPPLTSI-----IQVTPHRQGDRLILKATTMKSC---FKAERLHGRLRLSF--------------------------------------- 127
SlFAF1/2b  PPPLTSISGSERVRIRSHREGGRLVLRVVSINACNSYFKAERANGTLTLSW--RNNKVAQMDEEA------------------------- 163
SlFAF1/2c  PPPLTSMSGNEGVQIRPHREGGRLLLKATSITSWNSSFRVERANGRLKLSLLMHGEEDVIIQNDE------------------------- 205
SlFAF3/4a  PPPLTTIRGLNSVQFKPHREDGRLIIKAIEAPLTHNYLQAERSNGRLRLCFYKNEISASNIDENEELDEIEDETDIVIEEQEEEEEENTE 253
SlFAF3/4b  PPPLTTLRGSNSLQCRTYREGGRLVIKAIEAPKMRTYFHAERSNGRLTLCFLNDENSVSTIDEED--GESGDENDVFEREINDEEEEG-- 211

Consensus  -----------------N-----GE-G-----RP-RCKE-SG---K-F---W----WVAIS
SlFAF1/2a  -----------------NNTEILAQNG---YITPTKCNQ-NGTNIPTF--------WVAIS 159
SlFAF1/2b  ----------------NSCRKLASEIG-----IPRRCKEKSGSRIKGI-SSW-GQFWVAIF 201
SlFAF1/2c  ----------------ESCSSKLGEYS----SRPTRCKA-SGSRNKWI-SCW-EPFWVAIS 243
SlFAF3/4a  EKEEESDLYNVKRDMDGNKFDVEGEMGKEKCQRLSRCKE-SGKGNKVFGGDWRKALLVATS 313
SlFAF3/4b  -------MY-MKRDMNGNSHDVEGEIGIVNCQRVSRCKE-GRQGKKTF-CNWRNPLWVATS 262
```

Supplemental Figure 2. Sequences alignment of SlFAF proteins. The amino acid sequences of SlFAF1/2a, SlFAF1/2b, SlFAF1/2c, SlFAF3/4a and SlFAF3/4b were aligned using MUSCLE. The sequence of SlFAF proteins were compared to the consensus. The consensus threshold was set as >50%. Amino acids that match the reference are marked with gray highlighting.

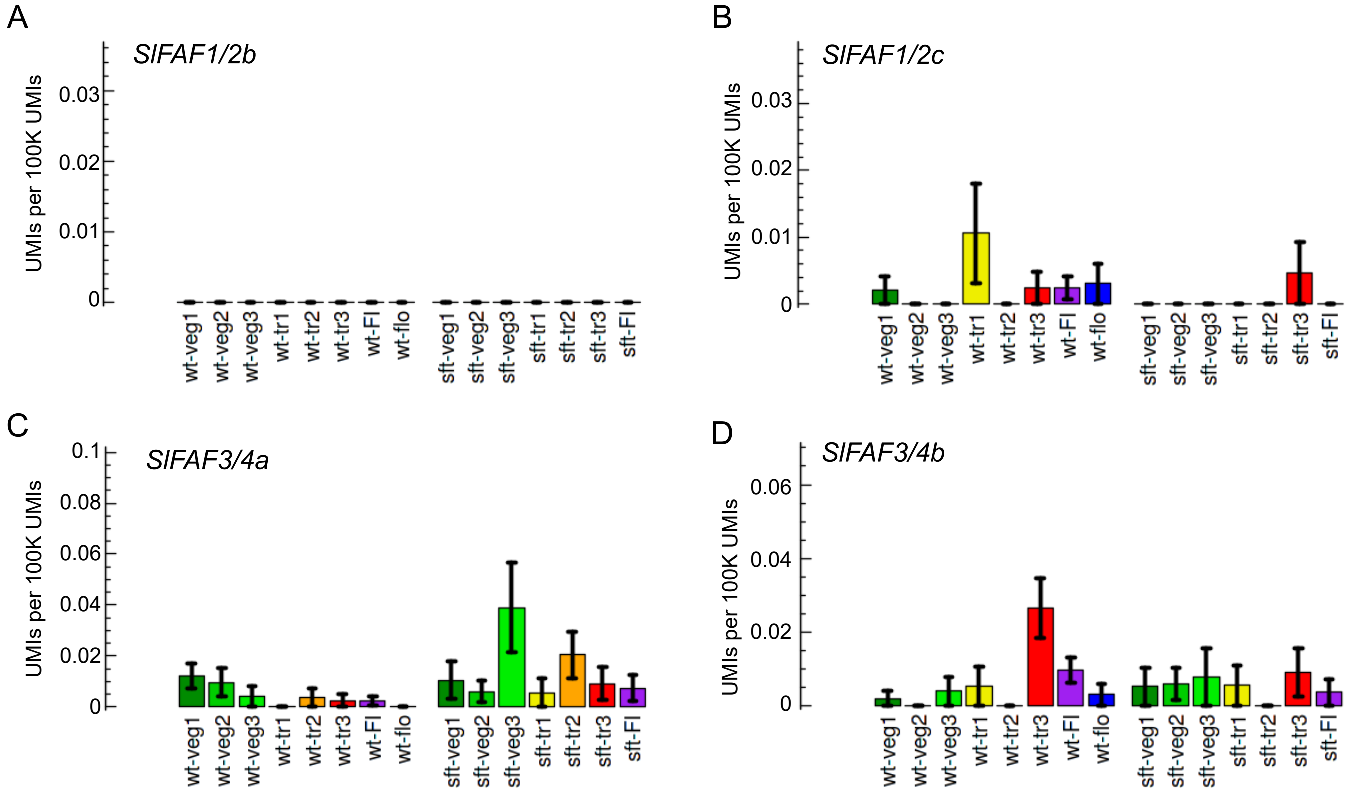

Supplemental Figure 3. Transcriptional dynamics of *SlFAF1/2b*, *SlFAF1/2c*, *SlFAF3/4a* and *SlFAF3/4b*. A-D, Expression levels of *SlFAF1/2b* (**A**), *SlFAF1/2c* (**B**), *SlFAF3/4a* (**C**) and *SlFAF3/4b* (**D**) in different SAM developmental phases from WT and the *sft* mutant. Vegetative phase was divided into three subphases (i.e., veg1-3). Three transition phases: tr1, tr2 and tr3. Flower initiation phase: FI. Floral meristem formed phase: flo. Expression levels on the y axis were computed as the unique molecular identifiers (UMIs) per 100,000 UMIs. The transcriptome data and graphs were automatically produced on the website: https://tanaylab.weizmann.ac.il/SMT/.

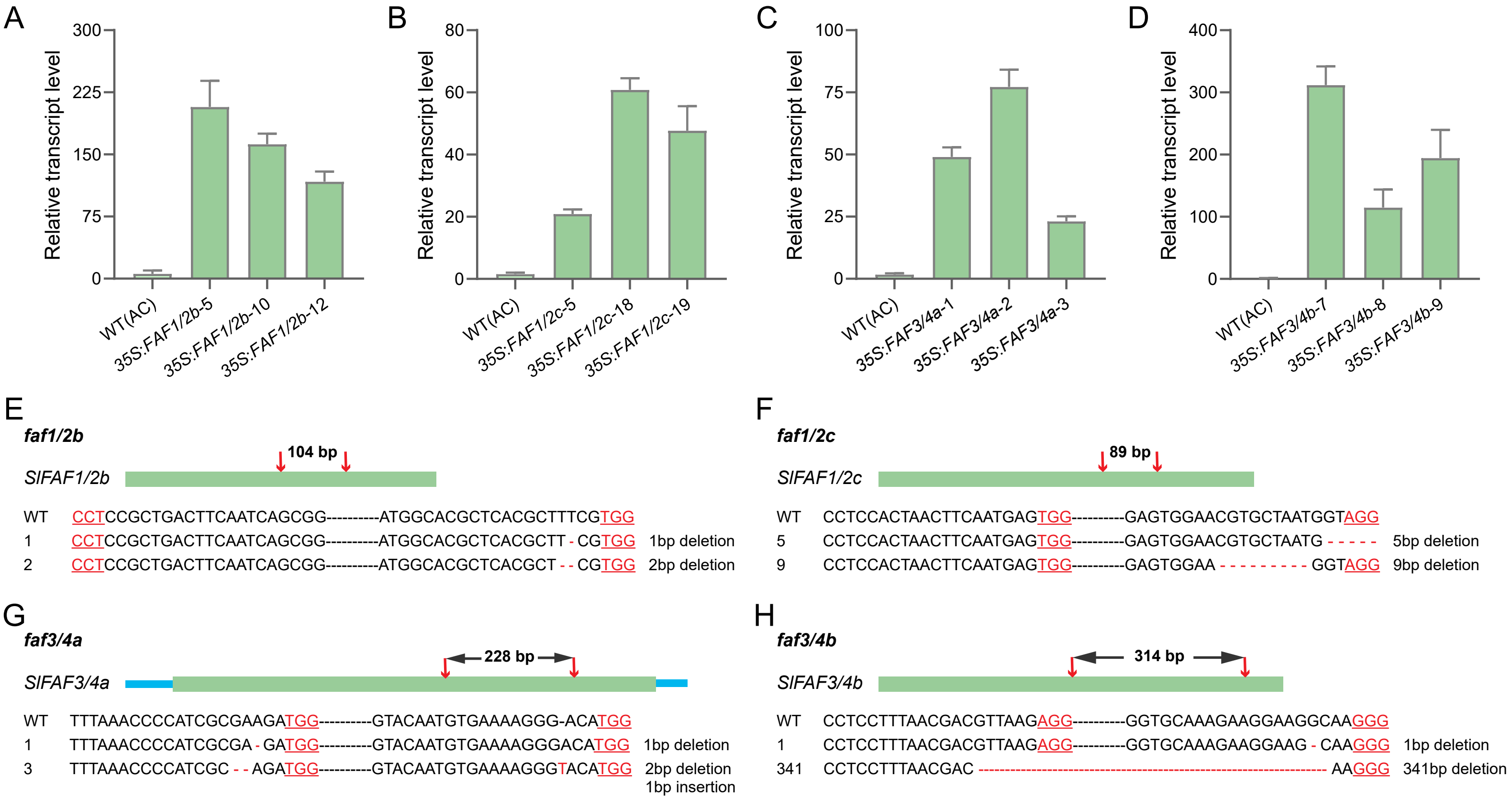

Supplemental Figure 4. Generations of *SlFAF1/2b*, *SlFAF1/2c*, *SlFAF3/4a* and *SlFAF3/4b* transgenic lines. A-D, Relative transcript levels of *SlFAF1/2b* (**A**), *SlFAF1/2c* (**B**), *SlFAF3/4a* (**C**) and *SlFAF3/4b* (**D**) in WT(AC) and their overexpressing lines. Three biological replicates were analyzed for each line. Error bars indicated SE. **E-H**, Generation of *faf1/2b*, *faf1/2c*, *faf3/4a* and *faf3/4b* mutants by CRISPR/Cas9. The sgRNAs sequences of *SlFAF1/2b*, *SlFAF1/2c*, *SlFAF3/4a* and *SlFAF3/4b* in the WT(AC) and in the *faf1/2b*-1 and *faf1/2b*-2 (**E**), *faf1/2c*-5 and *faf1/2c*-9 (**F**), *faf3/4a*-1 and *faf3/4a*-3 (**G**), *faf3/4b*-1 and *faf3/4b*-341 (**H**) mutants are shown.

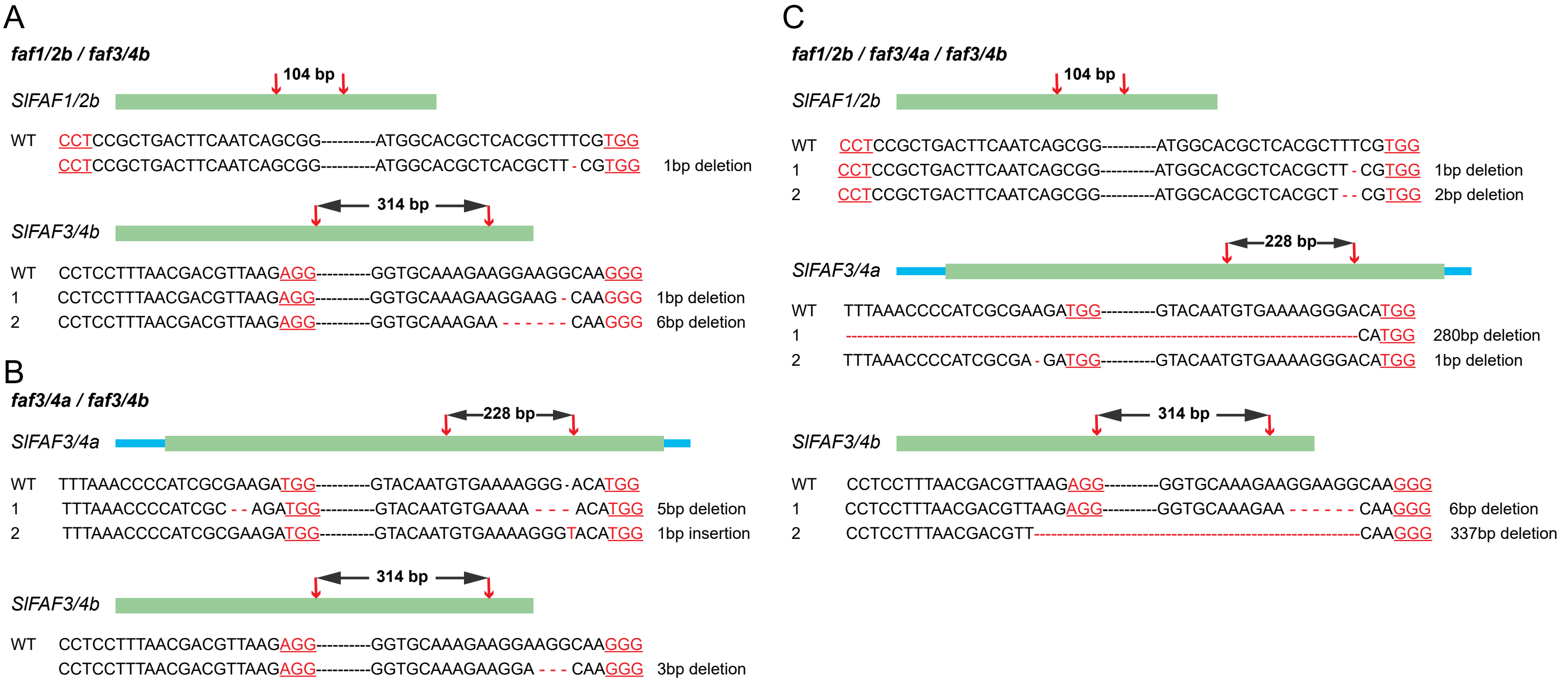

Supplemental Figure 5. Generations of double and triple *SlFAF* mutants using CRISPR/Cas9 system. A, *SlFAF1/2b* and *SlFAF3/4b* alleles in *faf1/2b faf3/4b* double mutants; sgRNAs sequences of *SlFAF1/2b* and *SlFAF3/4b* in WT (AC) and *faf1/2b faf3/4b*-1, *faf1/2b faf3/4b*-2. **B**, *SlFAF3/4a* and *SlFAF3/4b* alleles in *faf3/4a faf3/4b* double mutants; sgRNAs sequences of *SlFAF3/4a* and *SlFAF3/4b* in WT (AC) and *faf3/4a faf3/4b*-1, *faf3/4a faf3/4b*-2. **C**, *SlFAF1/2b*, *SlFAF3/4a* and *SlFAF3/4b* alleles in *faf1/2b faf3/4a faf3/4b* triple mutants; sgRNAs sequences of *SlFAF1/2b*, *SlFAF3/4a* and *SlFAF3/4b* in WT (AC) and *faf1/2b faf3/4a faf3/4b*-1, *faf1/2b faf3/4a faf3/4b*-2.

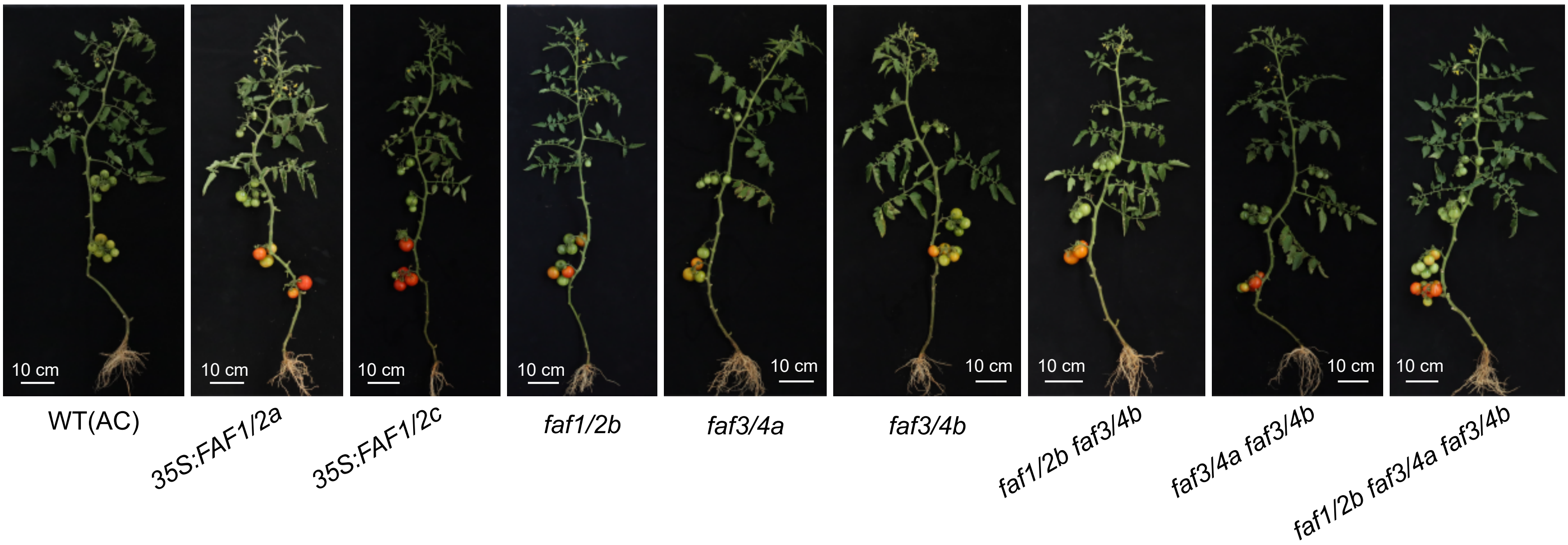

Supplemental Figure 6. Plants growth of early flowering *SlFAFs* overexpression and mutant lines. Plants from *35S: SlFAF1/2a, 35S: SlFAF1/2c*, CR-*faf1/2b, faf3/4a, faf3/4b* single mutant, *faf1/2b faf3/4b* and *faf3/4a faf3/4b* double mutant, *faf1/2b faf3/4a faf3/4b* triple mutant lines. Plants grown for about 110 days were used to investigate. Scale bar: 10 cm.

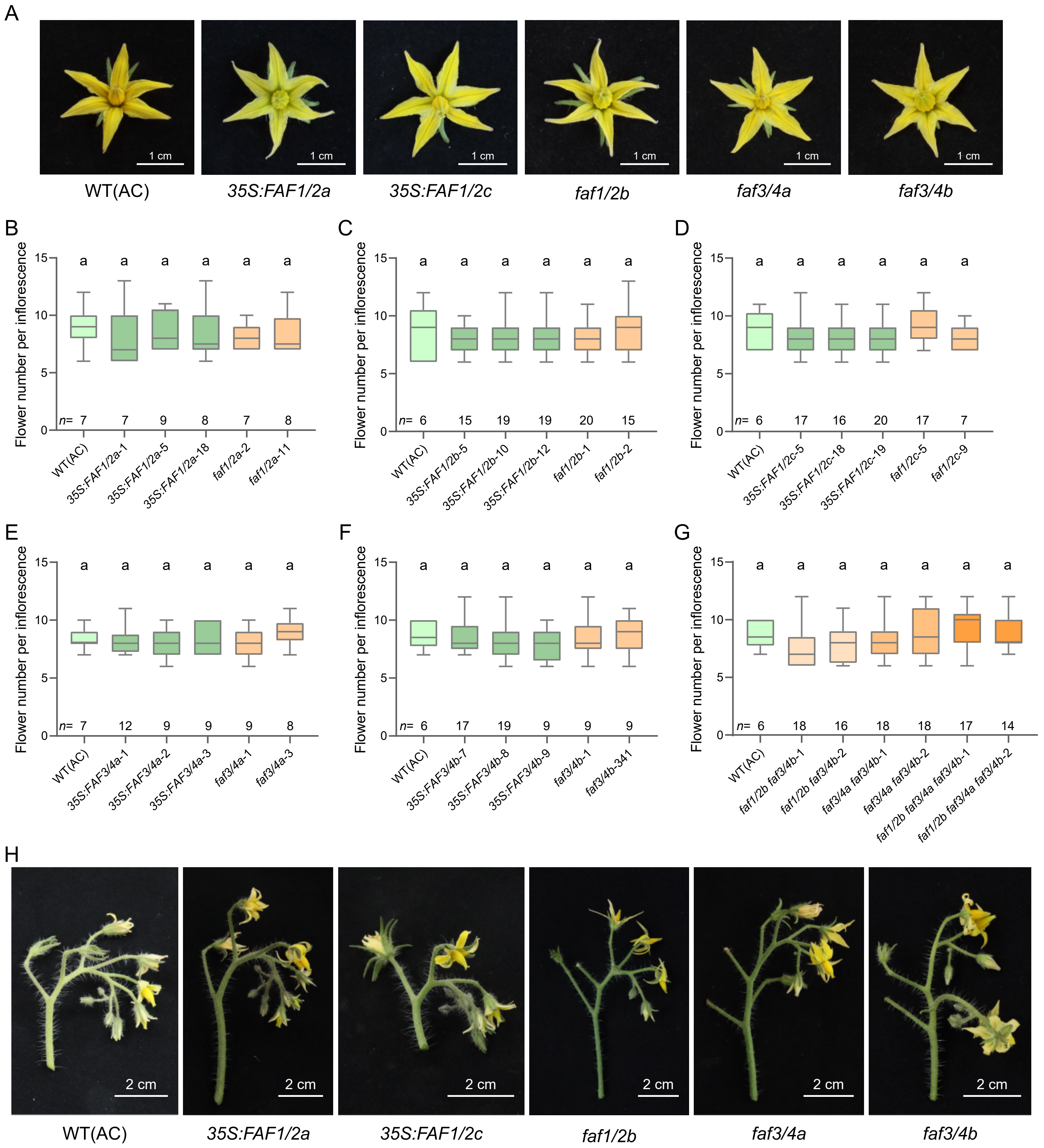

Supplemental Figure 7. Flower and inflorescence development of *SlFAFs* overexpression and mutant lines. A, Flowers from WT (AC), *35S: FAF1/2a*, *35S: FAF1/2c*, CR-*faf1/2b*, *faf3/4a* and *faf3/4b* mutants. Scale bar: 1 cm. **B-G,** Distribution of flower number per inflorescence in WT (AC) and *SlFAF1/2a* (**B**), *SlFAF1/2b* (**C**), *SlFAF1/2c* (**D**), *SlFAF3/4a* (**E**), *SlFAF3/4b* (**F**) overexpressing lines and single mutant lines, double and triple mutant lines (**G**). *n*, number of inflorescences investigated. **H,** Inflorescences from WT (AC), *35S: FAF1/2a*, *35S: FAF1/2c*, CR-*faf1/2b*, *faf3/4a* and *faf3/4b* mutants. Scale bar: 2 cm.

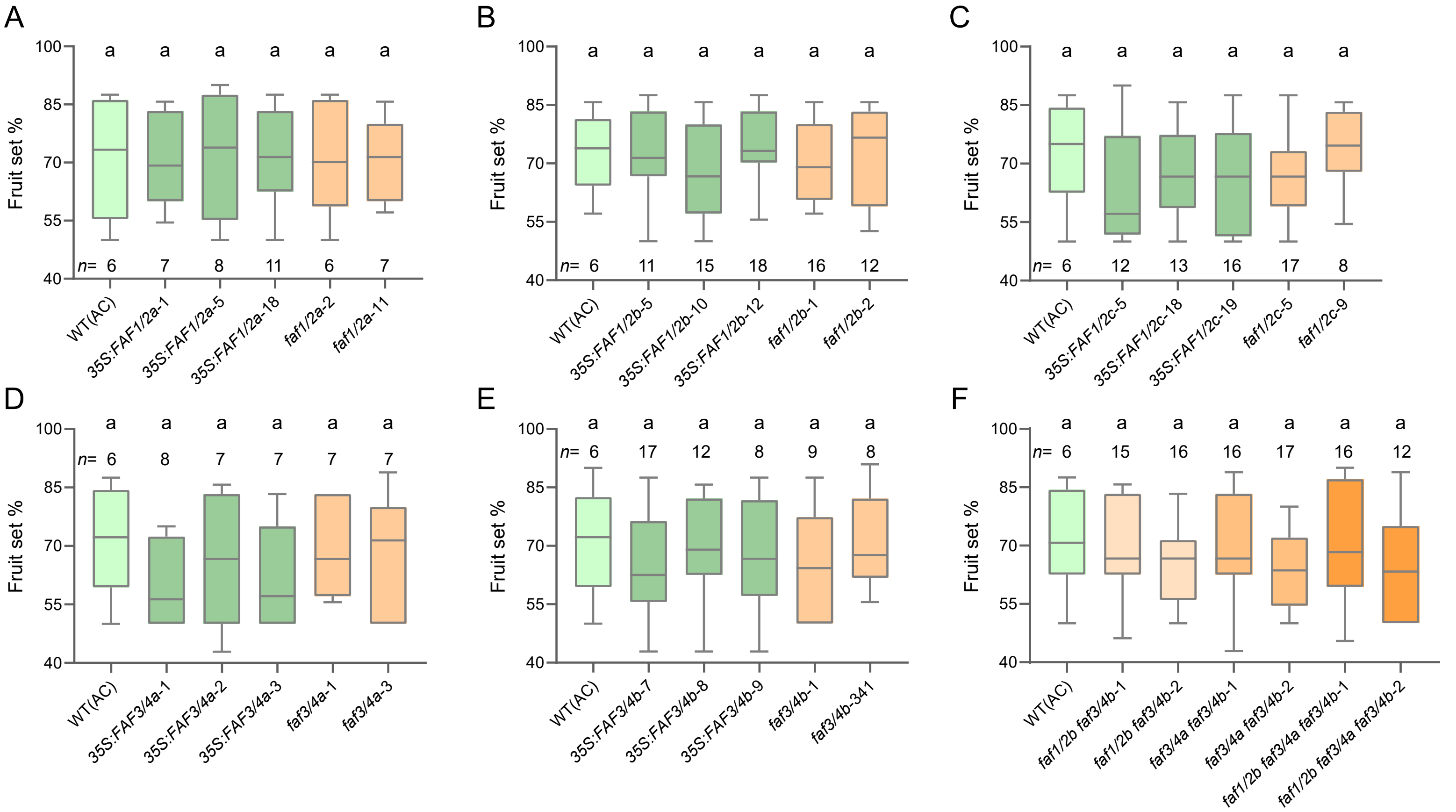

Supplemental Figure 8. Fruit set rates of *SlFAFs* overexpression and mutant lines. A-F, Distribution of fruit set rates in WT (AC) and *SlFAF1/2a* (**A**), *SlFAF1/2b* (**B**), *SlFAF1/2c* (**C**), *SlFAF3/4a* (**D**), *SlFAF3/4b* (**E**) overexpressing lines and single mutant lines, double and triple mutant lines (**F**). Fruit numbers were counted from the second or third inflorescence. *n*, number of inflorescences investigated.

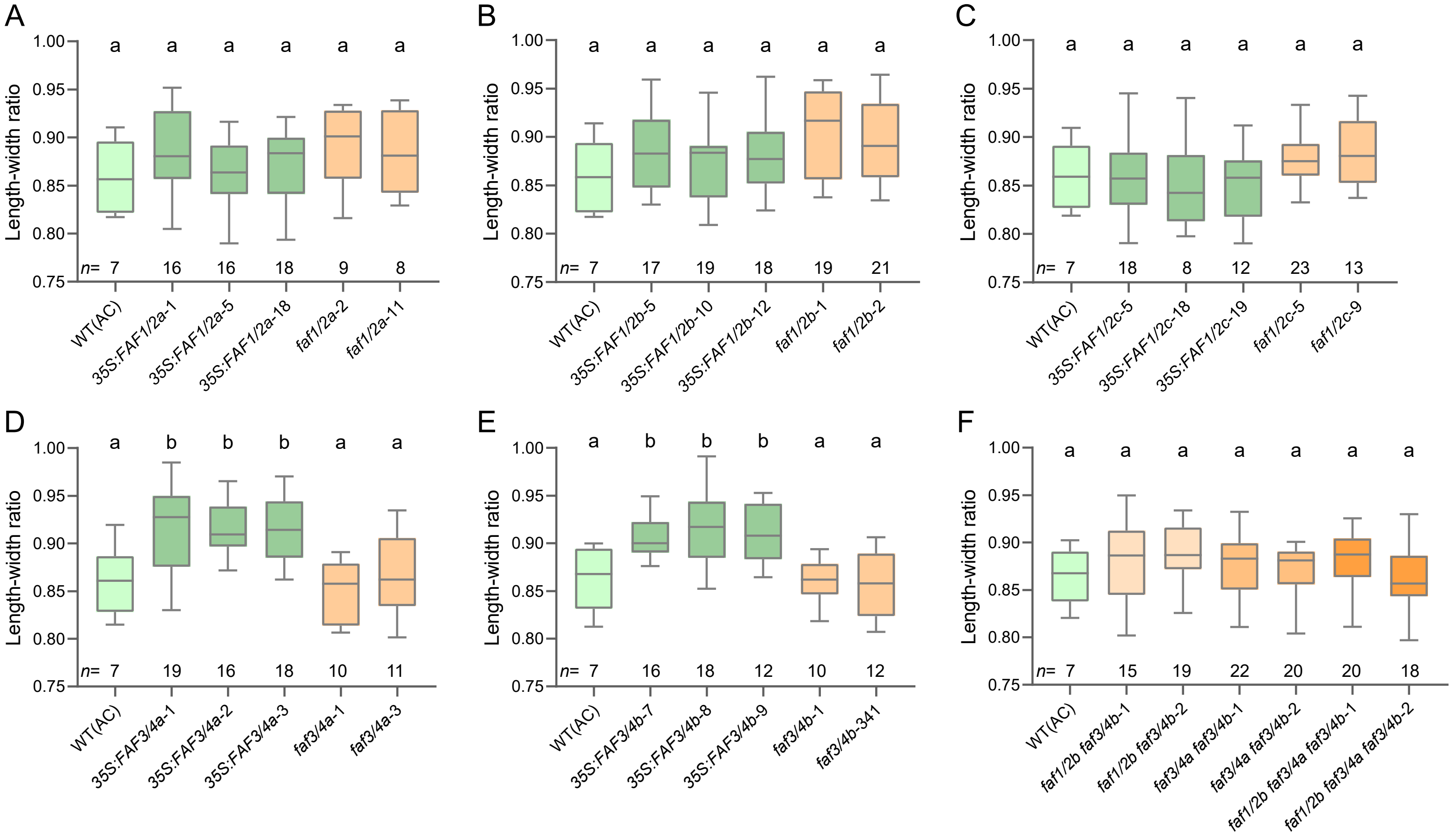

Supplemental Figure 9. Fruit shape of *SlFAFs* overexpression and mutant lines. A-F, Distribution of the ratio of fruit length to width in WT (AC) and *SlFAF1/2a* (**A**), *SlFAF1/2b* (**B**), *SlFAF1/2c* (**C**), *SlFAF3/4a* (**D**), *SlFAF3/4b* (**E**) overexpressing lines and single mutant lines, double and triple mutant lines (**F**). *n*, number of fruits investigated.
